# Unpacking bedaquiline heteroresistance: the importance of intermediate profiles for phenotypic drug susceptibility testing

**DOI:** 10.1128/aac.00356-25

**Published:** 2025-07-21

**Authors:** Nabila Ismail, Frik Sirgel, Shaheed Vally Omar, Shatha Omar, Marianna de Kock, Claudia Spies, Megan Folkerts, Grant Theron, Dave Engelthaler, John Metcalfe, Robin M. Warren

**Affiliations:** 1 South African Medical Research Council Centre for Tuberculosis Research, Division of Molecular Biology and Human Genetics, Faculty of Medicine and Health Sciences, Stellenbosch University 121470, Cape Town, South Africa; 2 Centre for Tuberculosis, National Institute for Communicable Diseases 70687, Johannesburg, South Africa; 3 Translational Genomics Research Institute (TGen) North Clinical Laboratory, Flagstaff, Arizona, USA; 4 University of California–San Francisco 8785, San Francisco, California, USA; University of Pittsburgh School of Medicine, Pittsburgh, Pennsylvania, USA

**Keywords:** *Mycobacterium tuberculosis*, bedaquiline, heteroresistance, phenotypic susceptibility testing

## Abstract

Phenotypic drug susceptibility testing (pDST) remains a widely used standard for determination of resistance for several drugs for the *Mycobacterium tuberculosis* complex. Next-generation sequencing technologies can identify heteroresistant populations at low frequencies, but little is known about the impact of heteroresistance on bedaquiline (BDQ) pDST results. We simulated heteroresistance using *in vitro*-generated *MmpR5* mutants mixed with the progenitor strain at various percentages (1%–20%) and performed pDST using the BACTEC MGIT 960 platform (1 and 2 µg/mL BDQ concentrations) coupled with EpiCenter TB-eXtended individual drug Susceptibility Testing software. Targeted next-generation sequencing was used to quantify the mutant subpopulation in growth control tubes, which were expected to maintain the mutant: wild-type proportion throughout the assay. Growth units of these growth control tubes were also comparable with minor differences in time to positivity between ratio mixtures. Only when intermediate results were considered (i.e., when growth units in a drug-containing tube reach the threshold for resistance but only after a further week of incubation) could BDQ heteroresistance be detected at frequencies of approximately 1% by pDST at a critical concentration of 1 µg/mL. These intermediate results, commonly disregarded during routine testing, could lead to earlier detection of BDQ resistance and may avert adverse clinical outcomes. The ability of pDST, a widely available DST technique, to reveal the presence of BDQ-resistant subpopulations at the phenotypic testing stage could improve resistance determination and potentially reduce time to effective treatment.

## INTRODUCTION

Multidrug-resistant tuberculosis (MDR-TB) is estimated to cause up to 19% of all antimicrobial resistance-attributable deaths worldwide. Furthermore, the success rate for the treatment of drug-resistant TB, in the presence of adherence, is poor at only 68%. The advent of bedaquiline (BDQ) has revolutionized MDR-TB care, but BDQ resistance determination is hampered by several obstacles facing both phenotypic and genotypic testing. This is despite the drug having received FDA approval for treatment of adults with pulmonary MDR-TB in 2012 (1).

Mutations in *MmpR5*, a gene encoding a transcriptional repressor (MmpR5), which downregulates transmembrane pump proteins MmpL5-S5, are the most common resistance-causing variants. These variants can be found across the length of the 498 bp gene, with no clear hotspot (2). Furthermore, in the 2023 WHO mutation catalog, novel loss-of-function (LoF) variants in *MmpR5* are graded as group 2 variants (i.e., “associated with resistance in the interim”), meaning further data are required to statistically support the association with resistance (3). A caveat to this rule, however, is that simultaneous LoF variants in MmpL5-S5 are epistatic (inhibit, mask, or suppress the impact of a LoF variant in *MmpR5*) and result in BDQ hypersusceptibility—a phenomenon detected primarily in the Lima, Peru region at a frequency of 43% in sublineage 4.11 isolates (4).

Of the three WHO-recommended targeted next-generation sequencing (tNGS) assays for determination of drug resistance, only one has met the class-based performance criteria for BDQ (5), but none cover the *mmpL5-S5* region (5). Additionally, the sensitivity for the use of tNGS to predict a resistant phenotype is currently only 68% for BDQ (6)—highlighting the continued necessity to rely on phenotypic testing to resolve any inadequacies. However, for phenotypic drug susceptibility testing (pDST), BDQ-resistant *Mycobacterium tuberculosis* isolates with variants in *MmpR5*, which may display minimal inhibitory concentration (MIC) values just below the critical concentration or are present at low frequencies (heteroresistance; the presence of mixed mutant and wild-type (wt) populations within a specimen), are often classed as susceptible. This may lead to the prescription of an ineffective regimen and amplification of resistance. In certain instances, variants in *MmpR5* (particularly those which result in incomplete repression of the MmpL5/S5 pump) can confer borderline resistant phenotypes (i.e., MIC values one dilution above or below the critical concentration)—the effect of which could be missed due to the technical variability of phenotypic testing (7, 8). Aside from the long turnaround time, the endorsed critical concentration for BDQ pDST using the MGIT 960 platform is based on limited evidence (7). A composite reference standard would be ideal to overcome the limitations of both phenotypic and genotypic BDQ susceptibility testing and can also be used to evaluate the significance of heteroresistance (7).

The impact of heteroresistance was highlighted in the 2023 WHO mutation catalog, when the inclusion of variants with an allele frequency below 75% increased the combined sensitivity of groups 1 and 2 variants (associated with resistance [interim]) by 10.2% and decreased the specificity by only 0.3% (3). In this same catalog, it was also considered that lowering the threshold of variant detection to 25% to account for lower-level BDQ heteroresistance improves prediction of a resistant phenotype (3). Here, we demonstrate a novel application of the BACTEC MGIT 960 platform coupled with EpiCenter TBeXiST (TB-eXtended individual drug Susceptibility Testing) software for phenotypic testing, which is currently the only routinely used method of BDQ pDST, to innovatively analyze phenotypic growth unit (GU) and time-to-positivity (TTP) data for early detection of BDQ resistance.

## MATERIALS AND METHODS

### Mutant and progenitor strains

**Fig 1:**
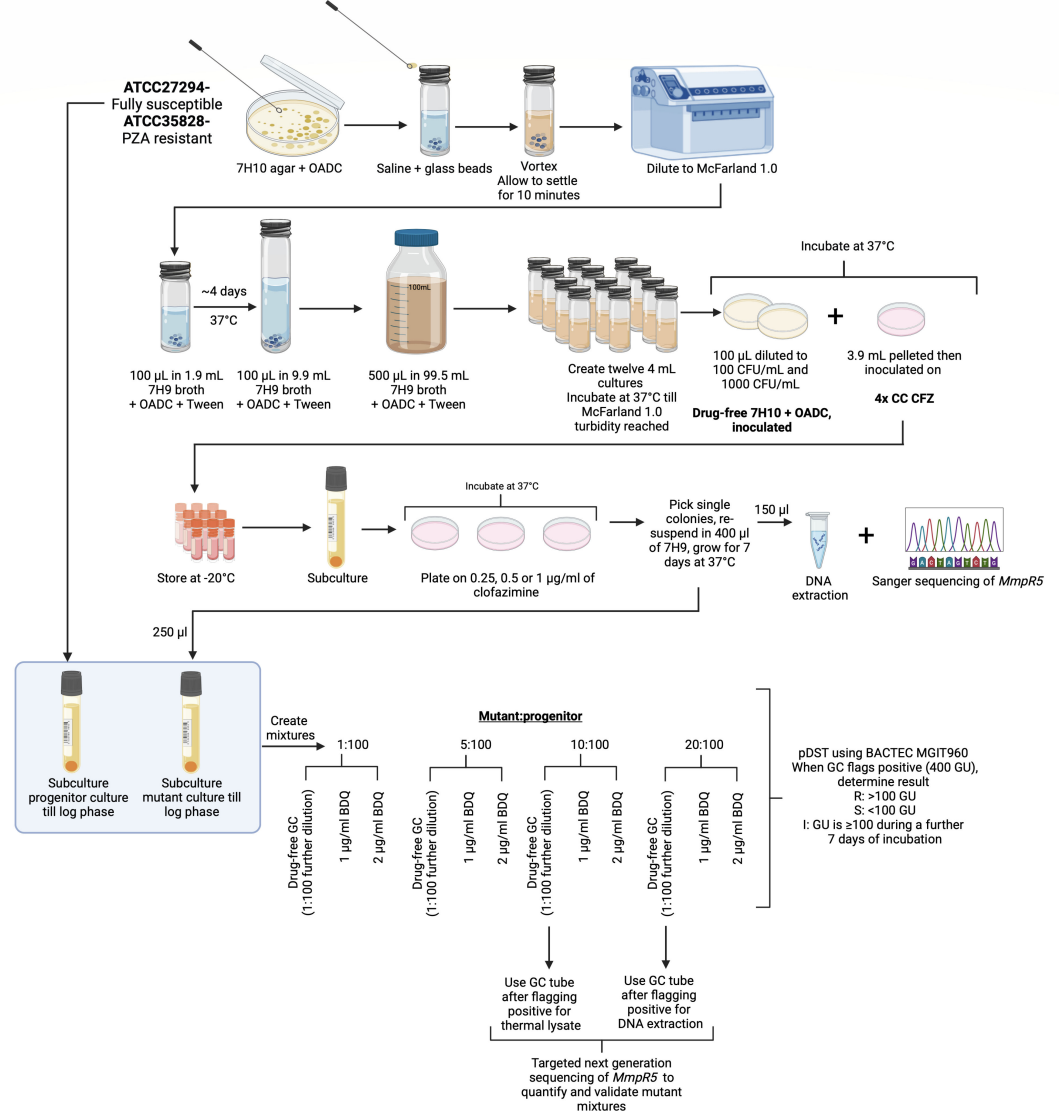
Method flow diagram for creation of clofazimine-resistant *in vitro* mutants, from which single colonies were picked and used for *MmpR5* Sanger sequencing. Pure mutant colonies were then grown to log phase and mixed with progenitor strains, also grown to log phase. Mutant strains are mixed at 1, 5, 10, 20: 100 ratios with progenitor strains. For each mixture, a drug-free control (GC), 1:100 dilution prepared as previously described (11, 12), and drug-containing test tubes (1 and 2 µg/mL BDQ) were set up. Resistant, susceptible, and intermediate pDST results were recorded from the TBeXiST software. Purified DNA and thermal lysates from growth from GC tubes (after flagging positive) for the 10 and 20% mixtures were used for *MmpR5* targeted sequencing to quantify and validate mixtures. Created in Biorender under agreement number JA28GLL9DE: https://BioRender.com/dquxqiq

*In vitro* mutants spontaneously generated with a Luria-Delbruck assay, using either an ATCC27294 (fully-susceptible H37Rv) or ATCC35828 (PZA-resistant) progenitor strain (BDQ MGIT 960 MIC values of 0.5 and 1 µg/mL), were selected on clofazimine (CFZ)-containing media as previously described (9). CFZ exposure is capable of generating *MmpR5* variants, which result in BDQ cross-resistance (10). We assumed that clofazimine mutants would likely have variants in *MmpR5*, which were not yet associated with bedaquiline resistance and thus be ideal candidates for the study. Mutants were further purified by selecting single colonies on BBL 7H11 agar base media (Becton Dickinson, NJ, USA) containing 0.25, 0.5, or 1 µg/mL of clofazimine (catalog no. A16462, AdooQ Biosciences, CA, USA) and supplemented with 10% (vol/vol) Middlebrook oleic acid, bovine albumin, sodium chloride, dextrose, and catalase (OADC) growth supplement (Becton Dickinson) and 0.5% (vol/vol) glycerol. Complete mutant characterization is in Table 1, an overview of the workflow is in Fig. 1 and detailed methods can be found in the supplemental material at https://github.com/NabilaIsmail/BDQhetero-supplementarydata.

**TABLE 1:** Genotypic and phenotypic data associated with each of the seven mutants as well as GUs for growth control (GC) tubes and percentage of mutant DNA from growth control tubes obtained using tNGS^*a*^

			Pure mutant		Mutant mixtures					
			MIC for 100% mutant (µg/mL)		Time taken for GC to reach 400 GU (hours)				% *MmpR5* variant detected with tNGS	
	Progenitor	*MmpR5* variant NT(AA)	BDQ MIC	CFZ MIC	1%	5%	10%	20%	DNA extracted from 20% GC tube	Thermal lysate from 10% GC tube
Mutant 1	ATCC35828	A97G (T33A)	>2.0	>2.0	187	174	179	162	20.52%	13.75%
Mutant 2	ATCC35828	G101T (R34L)	>2.0	>2.0	164	160	160	155	19.97%	10.27%
Mutant 3	ATCC27294	G126A (W42stop)	>2.0	2.0	229	198	206	232	23.3%	6.42%
Mutant 4	ATCC27294	192insG (fs)	>2.0	>2.0	182	197	171	193	30.12%	6.69%
Mutant 5	ATCC35828	193delG (fs)	>2.0	>2.0	Fail	168	179	184	9.64%	3.67%
Mutant 6	ATCC35828	G287C (R96P)	>2.0	>2.0	152	165	165	163	10.48%	5.81%
Mutant 7	ATCC35828	G326C (R109P)	>2.0	2.0	176	189	174	126	11.79%	8.33%

^*a*^ Two different progenitor strains were used for *MmpR5* mutant generation; ATCC27294 (BDQ MGIT 960 MIC: 0.5 µg/mL) and ATCC35828 (BDQ MGIT 960 MIC: 1 µg/mL). These progenitors were used to create corresponding mixtures. Each assay comprised a single GC tube and two drug-containing tubes (either 1 or 2 µg/mL BDQ). NT, nucleotide changes; AA, amino acid changes; fs, frameshift.

### Sanger sequencing

Each purified colony was inoculated into 400 µL MGIT medium (supplemented with OADC) and grown for 7 days at 37°C. An aliquot of this culture (250 µL) was inoculated into a fresh MGIT tube supplemented with 0.8 mL OADC (MGIT-OADC tube), while the remainder was used as a thermal lysate for PCR amplification of the *M. tuberculosis MmpR5* gene. Successfully amplified PCR products were submitted to the Central Analytical Facility of Stellenbosch University for post-PCR purification and Sanger sequencing. Sequencing chromatograms were visually inspected using BioEdit Sequence Alignment Editor software version 7.2.6 (13) to characterize *MmpR5* variants selected while ensuring there was no mix of wild-type with mutant sequences—confirming purification of the colonies.

### Drugs

BDQ (catalog no. A12327, AdooQ Biosciences, CA, USA) and CFZ were formulated in dimethyl sulfoxide ([DMSO], ref. 41639, Sigma-Aldrich Co.) to stock concentrations of 1 mg/mL and maintained at −70°C (max: 6 months).

### Minimum inhibitory concentration determination

The *in vitro*-generated mutants were subjected to MIC testing using a limited range of 0.5, 1, and 2 µg/mL for both BDQ and CFZ using the BACTEC MGIT 960 instrument, with results monitored on EpiCenter TBeXiST (Becton, Dickinson and Company, NJ, USA) as previously described (11, 12). When the GU of the drug-free GC reached 400, and if the GU of the drug-containing tube was ≥100, this was considered a resistant (R) result (11, 12). If the GU of the drug-containing tube was <100, the tube was incubated for a further 7 days. If the GU of the drug-containing tube was ≥100 during this further 7 days of incubation (after the GU of the drug-free control tube reached 400), the strain was intermediate (I) as previously described (14). If it was still <100, the strain was susceptible (S) (11, 12).

### Creation of heteroresistant cultures

Mutant strains for which BDQ and CFZ resistance was confirmed by pDST, and their corresponding progenitors (wt), were grown in MGIT-OADC tubes (Fig. 1) to the same time to positivity to ensure that both cultures entered the exponential growth phase when population mixtures were prepared. For each of the seven mutants, a set of four heteroresistant cultures was prepared with a mutant:wt ratio mix of approximately 1:100, 5:100, 10:100, and 20:100 corresponding to a 1%, 5%, 10%, or 20% subpopulation of a BDQ-resistant clone.

### Phenotypic drug susceptibility testing

For each of the heteroresistant cultures, a corresponding 1:100 GC was prepared in saline to represent 1% growth relative to the undiluted inocula. Five hundred microliters from this 1:100 dilution was transferred into the respective drug-free MGIT-OADC tubes. From each of the undiluted four heteroresistant mixtures, 0.5 mL was transferred into two MGIT-OADC tubes containing 1.0 µg/mL or 2.0 µg/mL BDQ. For quality control, undiluted wild-type or mutant strains were included to confirm their respective susceptibility and resistance when grown in BDQ-free and 1 µg/mL BDQ-containing MGIT-OADC tubes. A total of 28 assays were established (seven mutants at four different ratios) with a drug-free (GC) and two drug-containing tubes (test) for each assay (84 MGIT tubes in total, Fig. 1).

### Determination of pDST profile and analysis of growth units

pDST results were determined for each heteroresistant mixture by using the GU and TTP data obtained by the BACTEC MGIT 960 EpiCenter TBeXiST system as described above (11, 12). The time taken to reach 400 GU was also compared between all GC tubes for the various mixtures for each of the seven mutants, and minimal deviations were seen between time to positivity for growth in drug-free GC tubes with differing ratios of mutant:wt (Table 1). Following the determination of the pDST results, the entire volume from the 20% heteroresistant GC MGIT cultures (BDQ-free) was centrifuged, and the pellet was subjected to DNA extraction using the cetylrimethylammonium bromide (CTAB) DNA extraction method as previously described (15, 16). The entire volume from the 10% heteroresistant GC MGIT cultures (BDQ-free) was centrifuged, and the supernatant was removed, except for 500 µL. This remaining volume was then subjected to thermal lysis at 100°C for 40–60 minutes.

### Pilot study for heteroresistant culture quantification

To determine whether heteroresistant mixtures could be reproducibly created and quantified with tNGS, 5 mL of each of the heteroresistant mixtures (1%, 5%, 10%, or 20%) were created and were split into two sets (2.5 mL) (Fig. S1 at https://github.com/NabilaIsmail/BDQhetero-supplementarydata). Set A was subjected to thermal lysis (1.25 mL) and pDST (1.25 mL), and set B was subjected to DNA extraction (1.25 mL) and pDST (1.25 mL). Thus, DST was performed in duplicate (set A and set B). Thermal lysates (set A) or pure DNA (set B) from heteroresistant mixtures prior to DST (1%, 5%, 10%, and 20%) and the GC tubes post-DST (10% and 20%) were subjected to tNGS.

### Next-generation sequencing of heteroresistant cultures

Both thermal lysates and pure DNA were shipped to the Translational Genomics Institute North in Arizona (USA) and used for targeted next-generation sequencing of the *MmpR5* gene using a tiled, universal tailed method as previously described (17, 18). Data analysis was performed using the Amplicon Sequencing Analysis Pipeline with Single Molecule Overlapping Read technology as previously described (17, 18). Variants comprising at least 1% of each sample were reported using this software, which requires that forward and reverse sequencing reads agree to eliminate error. This ensures high confidence in variant calls. Variant frequencies were used to ensure that the dilutions created were in the expected range.

## RESULTS

### Mutant characterization

Seven purified mutant strains with *MmpR5* variants were selected for further use. These included four mutants (mutants 1, 2, 5, 6, and 7) derived from the ATCC35828 progenitor strain with *MmpR5* variants, g.A97G, g.G101T, g.193delG, g.G287C, and g.G326C, and two mutant strains (mutants 3 and 4) derived from ATCC27294 with *MmpR5* variants g.G126A and g.192insG (Table 1). All seven were determined to be CFZ-resistant (MIC values of 2 µg/mL) and cross-resistant to BDQ (MIC values >2 µg/mL). Mutant 3 with a nonsynonymous variant (g.G126A: p.W42stop) and mutants 4 and 5 with frameshift mutations in *MmpR5* (g.192insC or g.193delG) all possess WHO group 2 variants (3). Mutants 1, 2, 6, and 7 have nonsynonymous *MmpR5* variants resulting in amino acid substitutions: g.A97G(p.T33A); g.G101T(p.R34L) and g.G287C(p.R96P) are ungraded variants (reported but not graded), and g.G326C(p.R109P) is a WHO group 3 variant (i.e., having an uncertain association with BDQ resistance) (3).

### tNGS

tNGS served as a confirmatory assay to quantify mutant:wt ratios, thereby defining the input material. A pilot study (Fig. S1 at https://github.com/NabilaIsmail/BDQhetero-supplementarydata) comparing thermal lysates to DNA extracts showed that the 1% and 5% mixtures were too low for accurate detection by tNGS (Table S1 at https://github.com/NabilaIsmail/BDQhetero-supplementarydata). DST results were comparable when performed in duplicate. For further tNGS assays, thermal lysates from the 10% GC tube and pure DNA from the 20% GC tube were chosen as inputs as these were determined to effectively quantify mutant:wt ratios. Analysis of the tNGS data showed that mutant *MmpR5* DNA was detected at an average of 17% (range, 10%–30%) compared to the expected 20% from the drug-free GC (Table 1; Fig. S2 at https://github.com/NabilaIsmail/BDQhetero-supplementarydata). Thermal lysates from drug-free GC tubes showed an average of 7% (range, 4%–13%) of *MmpR5* mutant DNA compared to the expected 10% (Table 1; Fig. S2 at https://github.com/NabilaIsmail/BDQhetero-supplementarydata).

### Phenotypic drug susceptibility testing of heteroresistant mixtures

**Fig 2:**
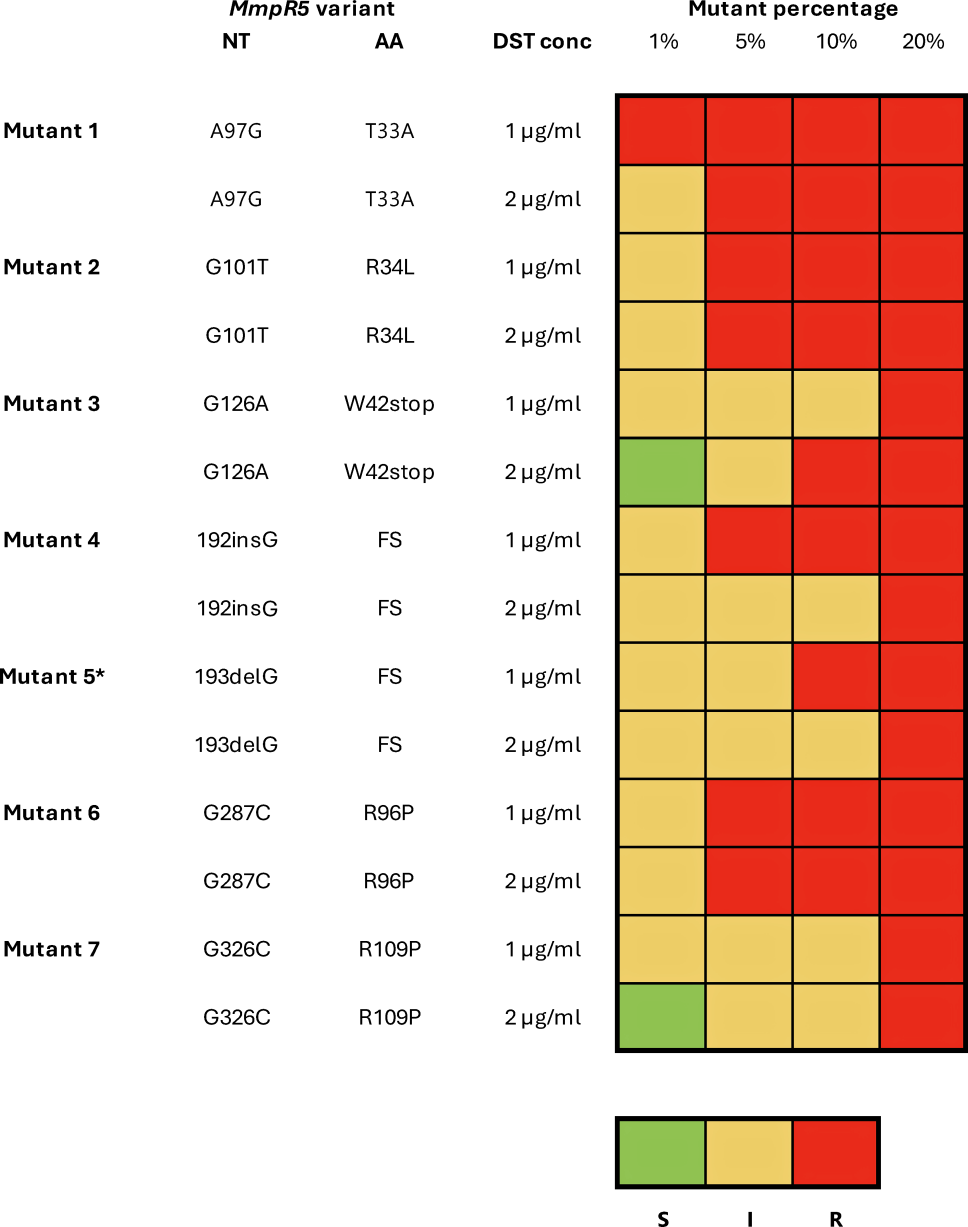
Heatmap showing the drug susceptibility profile at 1 and 2 µg/mL BDQ for each heteroresistant mixture at 1*, 5, 10, and 20% from seven mutants. Susceptible pDST results are shown in green (S), intermediate pDST results in yellow (I), and resistant pDST results in red (R). Nucleotide changes (NT) and amino acid (AA) changes are shown for each mutant. Mutants 1, 2 and 6 have *MmpR5* variants which have been previously described but are ungraded by the WHO. Mutants 3–5 have *MmpR5* variants which have a group 2 WHO grading (associated with BDQ resistance in the interim). Mutant 7 has a variant with a group 3 WHO grading, i.e., uncertain significance. *Mutant 5 DST result at 1% was determined using the GC from the 5% mutant as the TTP values were assumed to be similar, and the GC for the 1% mixture failed due to contamination.

Each mutant:wt mixture (5%, 10%, 20%, and 100%) underwent four distinct pDST assays (with corresponding drug-free GC, 1 µg/mL and 2 µg/mL BDQ-containing MGIT tubes, Fig. 1) with a single replicate per point. No variability could be assessed. The average time to positivity for the drug-free growth controls for all mixtures was 7.4 days (range [SD], 5.3–9.7 days [0.9 days]). At a 1 µg/mL concentration of BDQ, a resistant result was obtained from mutant 1 from the 1% mixture; from mutants 2, 4, and 6 from the 5% mixture; from mutant 5 from the 10% mixture; and from mutants 3 and 7 from the 20% mixture (Fig. 2). At lower ratios, mutants displayed an intermediate result (growth units in the drug-containing tube reached the threshold (≥100) for resistance but only after a further week of incubation) at 1 µg/mL of BDQ (Fig. 2; Table S2 at https://github.com/NabilaIsmail/BDQhetero-supplementarydata). At a 2 µg/mL concentration of BDQ, resistant results were obtained for mutants 1, 2, and 6 from the 5% mixture, for mutant 3 from the 10% mixture, and for mutants 4, 5, and 7 from the 20% mixture (Fig. 2). At lower ratios, the mutants displayed an intermediate result at 2 µg/mL of BDQ, except for mutants 3 and 7, which were susceptible from a 1% mixture (Fig. 2; Table S2 at https://github.com/NabilaIsmail/BDQhetero-supplementarydata).

**Fig 3:**
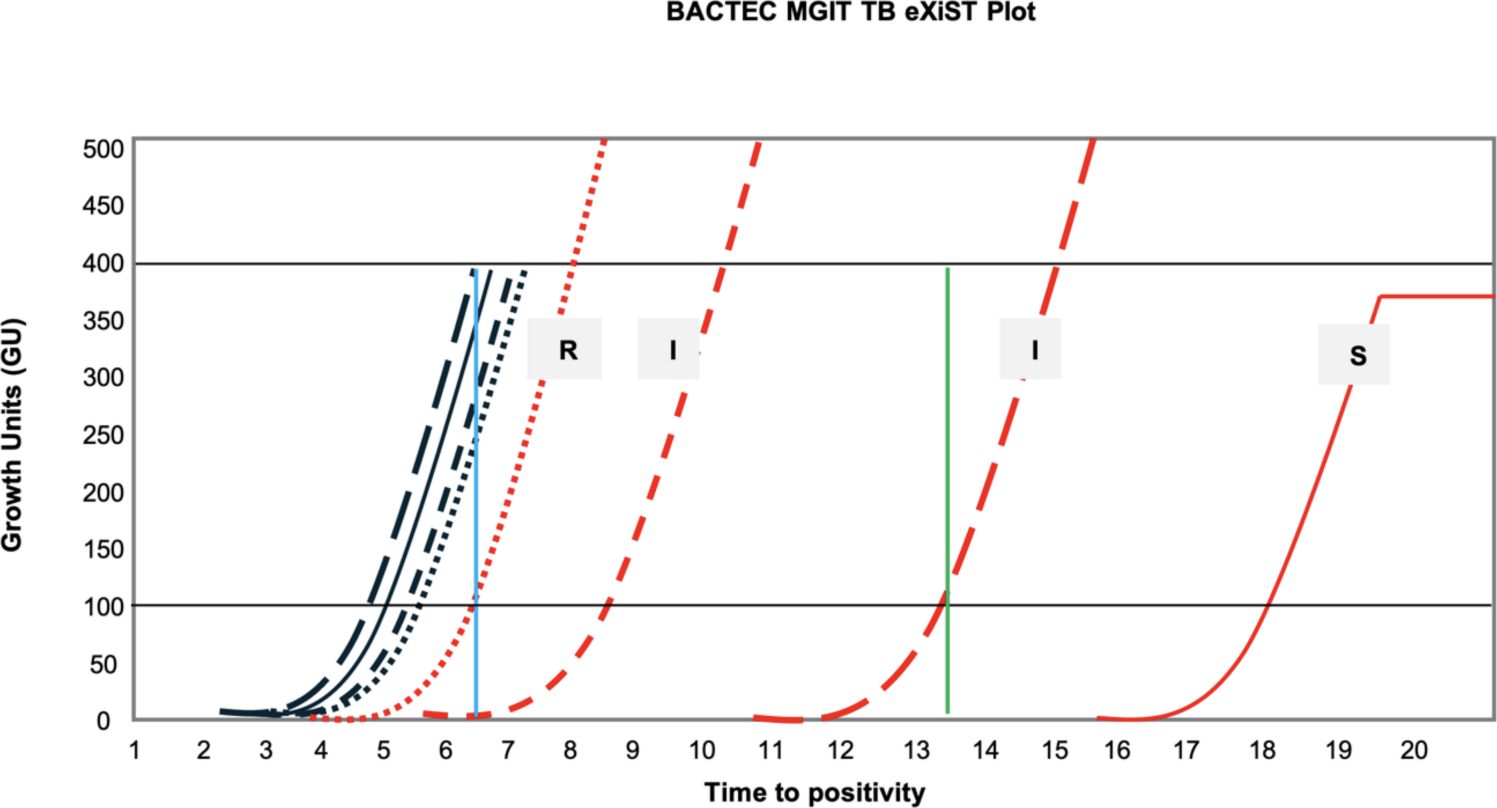
An example of a BACTEC MGIT 960 EpiCenter TBeXiST plot for the experimental set-up used. Drug-free growth controls are shown in black for 1% (—), 5% (— —), 10% (- - -), and 20% (**^…^**) heteroresistant mixtures with corresponding BDQ-containing tubes (1 µg/mL BDQ) in red (with susceptibility result labeled). Minimal deviation is seen between growth controls, as the *MmpR5* variant has no impact in the absence of BDQ (i.e., the variant has no fitness cost). Horizontal black lines indicate either 100 GUs (lower) or 400 GUs (upper), the relevant thresholds as defined previously (11, 12). The vertical blue line distinguishes the exact separation point between R and I results, which is dependent on when the GC tube flags at 400 GUs (in this figure, the 5% GC result is used, i.e., ~6.5 days). The vertical green line indicates the 7 day period after which the growth control has flagged 400 GUs (here, approximately day 14). Resistant (R), intermediate (I), or susceptible (S) results are reported when the corresponding GC has flagged positive at 400 GUs and up to 7 days after. R result: the drug-free growth control tube reaches 400 GUs, and the drug-containing tube is ≥100 GUs (11, 12). I result: the drug-free GC tube reaches 400 GUs while the drug-containing tube only reaches ≥100 during the further 7 days of incubation (14). S result: the drug-free GC tube reaches 400 GUs, and the drug-containing tube remains <100 following 7 further days of incubation (11, 12). The plateau of the final exponential curve is indicative of the assay being ended in the instrument.

Figure 3 clearly highlights the importance of the intermediate category. The DST technique measures 99% inhibition of bacterial numbers to differentiate between resistance and susceptibility. Since resistant mutants were used for the preparation of the mixtures, the results show that at 2 µg/mL, we may fail to detect some of the resistant subpopulations in certain instances (e.g., mutants 3 and 7 which were susceptible from a 1% mixture). However, in most instances, using the current critical concentration (1 µg/mL) and the intermediate category, we can detect these resistant populations from as low as 1%. Furthermore, even without the use of the intermediate category, at 1 µg/mL, we detected heteroresistant populations from as low as 5% in more than half the cases. Additionally, the intermediate results obtained for these mutants cross the threshold for resistance (GU ≥100) as early as 3 hours and up to 133 hours or 5.5 days after the corresponding GC has flagged positive (Table S2 at https://github.com/NabilaIsmail/BDQhetero-supplementarydata).

## DISCUSSION

Following 60 years of use, pDST is still implemented globally as a reflex standard-of-care assay for several drugs. In this study, we show how the routinely used BACTEC MGIT 960 platform coupled with EpiCenter TBeXiST software can detect BDQ-resistant subpopulations using available GU data. Through the extension of the incubation period of DST assays for a further 7 days, intermediate results can be determined. Differences in the BDQ resistance profile for several mutants displaying a variety of *MmpR5* variants (including novel and group 3 variants) were observed using the MGIT 960 platform and two BDQ concentrations.

We attempted to explore the boundaries of the widely implemented MGIT 960 assay to detect minor BDQ-resistant subpopulations through the creation of low-level heteroresistant mixtures. While the MGIT 960 assay is a qualitative assay exploiting fluorescence of an oxygen sensor for determination of inhibition of growth or lack thereof, coupling with output from the EpiCenter TBeXiST makes quantitative pDST possible. We used several additional quantitative methods to validate our results and to ensure reproducibility for each of the seven mutants. This included dilution of a pure mutant culture to approximately 1, 5, 10, and 20%; tNGS from thermal lysates and pure DNA coupled with GU data; and individual assays for each mutant at two different BDQ concentrations to maximize scientific rigor.

To simplify resistance classification, particularly in high-burden settings, a binary classification is used for MGIT 960 results, i.e., either “S” for susceptible or “R” for resistant (19). Current binary classification methods categorize intermediate results as susceptible due to the 1% proportion rule, limiting the detection of minor resistant subpopulations. By extending the incubation period of MGIT 960 DST assays by 7 days, we were able to capture intermediate results indicative of heteroresistant populations. This can be achieved through interpretation of growth curve data (Fig. 3), easily acquired from the BACTEC MGIT 960 EpiCenter TBeXiST system which is universally used in conjunction with the MGIT 960 (11, 12). It should be clarified that intermediate in this study is referring to the presence of <1% growth rather than a resistance category falling between susceptible and resistant categories. The category “intermediate” was used at the time of creation of the EpiCenter software (described in detail by Springer, B. et al. [14]). Therefore, heteroresistant populations should be easily detectable within the routine setting, with data on susceptibility as well as any evidence of minor resistant populations within a week, without drastic changes made to current testing algorithms. This method allows for more nuanced resistance profiling, offering a means to detect emerging resistance. Whether these resistant subpopulations are clinically significant for BDQ treatment does warrant further investigation. However, a study by Colangeli et al*.* (20) showed that elevated isoniazid or rifampicin MIC values (below clinical breakpoints) were associated with greater risk of relapse in pretreatment isolates.

The current MGIT 960 breakpoint for BDQ was established based on limited data and remains a matter of contention (7, 21), coupled with variants which have borderline MICs and the technical variability of phenotypic testing (7); BDQ pDST faces multiple obstacles. To account for these challenges, we also made use of an additional concentration, 2 µg/mL BDQ, to enable a more granular interpretation of the resistance profiles for various heteroresistant mixtures. Although the phenotypic heterogeneity of underlying resistant populations is readily observed through growth curve data, the use of this higher concentration also shows that a limit of detection suitable to identify heteroresistance could be based on criteria that are different from those used for pDSTs, i.e., a higher concentration could differentiate heteroresistant populations with greater clarity. Importantly, we used mutants with variants across the spectrum of the WHO grading criteria, i.e., LoF or frameshift variants, reported but ungraded variants, and variants with uncertain association with BDQ resistance (3). The use of the intermediate category for identification of heteroresistant populations proved to be valuable in all instances.

Several limitations exist when working with *M. tuberculosis* cultures. First, the bacteria are prone to clump in liquid MGIT culture with a “breadcrumb” appearance (22). We addressed this as best as possible with the creation of uniform mixtures through vortexing, allowing the mixtures to settle and using the supernatant. Second, due to the slow-growing nature of *M. tuberculosis*, a 24 hour difference in the time to positivity of the growth control tubes would represent a change equivalent to half of the bacterial population, and this variability can be observed between GC tubes (Fig. S2 at https://github.com/NabilaIsmail/BDQhetero-supplementarydata). Finally, it appears that not all mutants return the same resistance profile; this could be due to differential growth in the mutant:wt mixture ratios (exhibited in differing tNGS percentages, [Fig. S2 at https://github.com/NabilaIsmail/BDQhetero-supplementarydata]) or by the fact that not all *MmpR5* variants are equally responsible for high-level resistance (23). This may also be attributed to the degree by which specific *MmpR5* variants increase efflux pump activity and cause a reduction in the effective intracellular BDQ concentration. These possible scenarios were overcome using *in vitro*-generated mutants, with *MmpR5* variants all associated with phenotypic resistance (i.e., an MIC >1 µg/mL), as well as by using mutant and progenitor cultures grown to the same log phase prior to heteroresistant mixtures being created.

Heteroresistance, characterized by mixed mutant and wild-type populations, is increasingly recognized as a challenge in TB treatment. In the case of BDQ heteroresistance, several studies have used next-generation sequencing to show the clinical impact of low-frequency variants in *MmpR5* (24–27). Previous studies have shown that in the absence of a supporting regimen to prevent resistance acquisition, *MmpR5* variants over time may lead to phenotypic resistance and poor treatment outcomes (28–31); an intermediate result would presumably have similar associations in this context. Ideally, an NGS technology utilized directly on clinical specimens and capable of detecting *MmpR5* and concurrent *MmpL5-S5* variants below 25% should be the reflex test for determination of BDQ resistance. If a variant with an uncertain association is identified, pDST should be repeated or MIC should be performed using an inoculum from the intermediate MGIT tube. This could result in an “R” pDST result, confirming the presence of heteroresistant populations (32) or elevated MIC values. The use of this composite reference standard would allow variants to be contributed to the WHO for the update of the mutation catalog, as well as improve time to detection of resistance. In summary, the ability to identify heteroresistant populations using existing diagnostic tools has important implications for TB resistance surveillance and treatment strategies. Future studies should focus on validating these findings with clinical samples and integrating them into routine DST workflows to improve early detection and patient outcomes.
